# Multisignal control of expression of the LHCX protein family in the marine diatom *Phaeodactylum tricornutum*

**DOI:** 10.1093/jxb/erw198

**Published:** 2016-05-25

**Authors:** Lucilla Taddei, Giulio Rocco Stella, Alessandra Rogato, Benjamin Bailleul, Antonio Emidio Fortunato, Rossella Annunziata, Remo Sanges, Michael Thaler, Bernard Lepetit, Johann Lavaud, Marianne Jaubert, Giovanni Finazzi, Jean-Pierre Bouly, Angela Falciatore

**Affiliations:** ^1^Sorbonne Universités, UPMC, Institut de Biologie Paris-Seine, CNRS, Laboratoire de Biologie Computationnelle et Quantitative, 15 rue de l’Ecole de Médecine, 75006 Paris, France; ^2^Department of Biotechnology, University of Verona, Strada Le Grazie, I-37134 Verona, Italy; ^3^Institute of Biosciences and BioResources, CNR, Via P. Castellino 111, 80131 Naples, Italy; ^4^Biology and Evolution of Marine Organisms, Stazione Zoologica Anton Dohrn, Villa Comunale, 80121 Naples, Italy; ^5^Institut de Biologie Physico-Chimique, UMR 7141 CNRS-UPMC, 13 rue Pierre et Marie Curie, 75005 Paris, France; ^6^Zukunftskolleg, Department of Plant Ecophysiology, University of Konstanz, D-78457 Konstanz, Germany; ^7^UMI 3376 TAKUVIK, CNRS/Université Laval, Département de Biologie, Pavillon Alexandre-Vachon, 1045 avenue de la Médecine, Québec (Québec) G1V 0A6, Canada; ^8^Laboratoire de Physiologie Cellulaire et Végétale, UMR 5168, Centre National de la Recherche Scientifique (CNRS), Institut National Recherche Agronomique (INRA), Université Grenoble Alpes, Commissariat à l’Energie Atomique et aux Energies Alternatives (CEA), Institut de Biosciences et Biotechnologies de Grenoble, (BIG), CEA Grenoble, F-38054 Grenoble cedex 9, France

**Keywords:** Dark, gene expression, iron starvation, LHCX, light, marine diatom, nitrogen starvation, non-photochemical quenching.

## Abstract

Diatoms are phytoplanktonic organisms that grow successfully in the ocean where light conditions are highly variable. Studies of the molecular mechanisms of light acclimation in the marine diatom *Phaeodactylum tricornutum* show that carotenoid de-epoxidation enzymes and LHCX1, a member of the light-harvesting protein family, both contribute to dissipate excess light energy through non-photochemical quenching (NPQ). In this study, we investigate the role of the other members of the LHCX family in diatom stress responses. Our analysis of available genomic data shows that the presence of multiple *LHCX* genes is a conserved feature of diatom species living in different ecological niches. Moreover, an analysis of the levels of four *P. tricornutum* LHCX transcripts in relation to protein expression and photosynthetic activity indicates that LHCXs are differentially regulated under different light intensities and nutrient starvation, mostly modulating NPQ capacity. We conclude that multiple abiotic stress signals converge to regulate the LHCX content of cells, providing a way to fine-tune light harvesting and photoprotection. Moreover, our data indicate that the expansion of the *LHCX* gene family reflects functional diversification of its members which could benefit cells responding to highly variable ocean environments.

## Highlight

Multiple stress signalling pathways regulate *LHCX* family gene expression in the diatom *Phaeodactylum tricornutum* to attune acclimation responses efficiently in highly variable ocean environments.

## Introduction

The perception of environmental signals and the activation of appropriate responses to external stimuli are of major importance in the growth and survival of all organisms. At the cellular level, this requires the presence of complex signal perception and transduction networks, triggering changes in nuclear gene expression (Lee and Yaffe, 2014). External cues such as light, temperature, and nutrient availability strongly affect the physiology and metabolism of photosynthetic organisms, so acclimation mechanisms are needed to cope efficiently with short- and long-term environmental changes to maintain photosynthetic performances (Walters, 2005; Eberhard *et al.*, 2008). In eukaryotic phototrophs, chloroplast biogenesis and activity are integrated in broader regulatory programmes, requiring coordination between the nucleus and chloroplast genomic systems (Rochaix, 2011; Jarvis and Lopez-Juez, 2013). The nucleus responds to stimuli inducing the synthesis of regulatory proteins that modulate chloroplast responses. In turn, molecules originating from the chloroplast activity (e.g. redox state of the photosynthetic electron carriers, reactive oxygen species, plastid gene transcription, tetrapyrroles, and other metabolites) provide a retrograde signal feeding back to the nucleus (Woodson and Chory, 2008).

Marine photosynthesis is dominated by unicellular phytoplanktonic organisms, which are passive drifters in the water column and often experience drastic changes in their surrounding environment (Falkowski *et al.*, 2004; Depauw *et al.*, 2012). Diatoms are among the most abundant and diversified groups of photosynthetic organisms. They are particularly adapted to growing in very dynamic environments such as turbulent coastal waters and upwelling areas, as well as in polar oceans (Margalef, 1978; Field *et al.*, 1998; Kooistra *et al.*, 2007; Arrigo *et al.*, 2012). Several species can survive for long periods at depths where light is limiting for growth, and quickly reactivate their metabolism after returning to the photic zone (Sicko-Goad *et al.*, 1989; Reeves *et al.*, 2011). The adaptive capacity of such algae suggests that they have sophisticated mechanisms to perceive and rapidly respond to environmental variations. Consistent with this notion, genome sequence information of representative diatom model species such as *Thalassiosira pseudonana* and *Phaeodactylum tricornutum* (Armbrust *et al.*, 2004; Montsant *et al.*, 2007; Bowler *et al.*, 2008; Rayko *et al.*, 2010), and the availability of transcriptomic and proteomic data in various species exposed to different stimuli and stresses (Nymark *et al.*, 2009; Dyhrman *et al.*, 2012; Thamatrakoln *et al.*, 2012; Ashworth *et al.*, 2013; Nymark *et al.*, 2013; Keeling *et al.*, 2014; Valle *et al.*, 2014; Alipanah *et al.*, 2015; Muhseen *et al.*, 2015) have highlighted the existence of some diatom-specific adaptive strategies, pinpointing molecular regulators of environmental change responses. Several photoreceptors for efficient light colour sensing (Huysman *et al.*, 2013; Schellenberger Costa *et al.*, 2013; Fortunato et al., 2015, 2016) have been identified in diatoms. Peculiar iron acquisition and concentration mechanisms are also known (Allen *et al.*, 2008; Marchetti *et al.*, 2012; Morrissey *et al.*, 2015), which contribute to their survival in iron-limited waters and to their rapid proliferation when iron becomes available (de Baar *et al.*, 2005). Diatoms have peculiar gene sets implicated in nitrogen metabolism, such as a complete urea cycle, that could be used as temporary energy storage or as a sink for photorespiration (Allen *et al.*, 2011). Eventually, diatoms optimize their photosynthesis via extensive energetic exchanges between plastids and mitochondria (Bailleul *et al.*, 2015).

The ecological dominance of diatoms also relies on their capacity to cope with light stresses, thanks to very efficient photoprotective mechanisms. Diatoms possess a high capacity to dissipate excess light energy as heat through high energy quenching (qE) that, together with the photoinhibitory quenching (qI), can be visualized via the non-photochemical quenching (NPQ) of Chl *a* fluorescence (Lavaud and Goss, 2014; Goss and Lepetit, 2015). The xanthophyll diatoxanthin (Dt) pigment, synthesized from the de-epoxidation of diadinoxanthin (Dd) during illumination (Goss and Jakob, 2010; Lavaud *et al.*, 2012), and the LHCX1 protein, a member of the light-harvesting protein family (Bailleul *et al.*, 2010), have been identified as key components of the qE process in diatoms. *P. tricornutum* cells with deregulated LHCX1 expression display a significantly reduced NPQ capacity and a decreased fitness, demonstrating a key role for this protein in light acclimation (Bailleul *et al.*, 2010), similarly to the light harvesting complex stress-related (LHCSR) proteins of green algae and mosses (Alboresi *et al.*, 2010; Ballottari *et al.*, 2016).

Multiple nuclear-encoded and plastid-localized LHCX family members have been identified in the genomes of the diatoms *P. tricornutum* and *T. pseudonana*. Scattered information derived from independent gene expression analyses indicated that some *LHCX* isoforms are constitutively expressed while others are expressed in response to stress (Becker and Rhiel, 2006; Allen *et al.*, 2008; Nymark *et al.*, 2009; Zhu and Green, 2010; Bailleul *et al.*, 2010; Beer *et al.*, 2011; Lepetit *et al.*, 2013), similarly to what is observed for the two LHCSR proteins in *Physcomitrella patens* (Gerotto *et al.*, 2011). In this study, we have extended the characterization of the four *P. tricornutum* LHCXs, by combining detailed gene expression analysis in cells exposed to different conditions with *in vivo* analysis of photosynthetic parameters. The result of this analysis revealed a complex regulatory landscape, suggesting that the expansion of the LHCXs reflects a functional diversification of these proteins and may contribute to the regulation of the chloroplast physiology in response to diverse extracellular and intracellular signals.

## Materials and methods

### Analysis of the *LHCXs* in the diatom genomes

*Phaeodactylum tricornutum* and *T. pseudonana* *LHCX* gene model identifiers were retrieved from the diatom genomes, respectively, on *P. tricornutum* Phatr2 and *T. pseudonana* Thaps3 in the JGI database (http://genome.jgi.doe.gov/). *Phaeodactylum tricornutum* LHCX proteins were used as query to perform BlastP searches on the *Pseudo-nitzschia multiseries* (http://genome.jgi.doe.gov/Psemu1/Psemu1.home.html) and *Thalassiora oceanica* (http://protists.ensembl.org/Thalassiosira_oceanica/Info/Index) genome portals. Best hit sequences were tested on Pfam (http://pfam.xfam.org/) to assess the presence of the Chloroa_b-bind domain (PF00504), characteristic of light-harvesting proteins. Protein alignments were performed with MUSCLE (http://www.ebi.ac.uk/Tools/msa/muscle/).

### Diatom growth conditions

The *P. tricornutum* (Pt1 8.6, CCMP2561) cultures, obtained from the Provasoli-Guillard National Center for Culture of Marine Phytoplankton, were used for the gene expression and photophysiology analyses. Cells were grown in ventilated flasks in f/2 medium (Guillard, 1975) at 18 °C, in a 12h light/12h dark photoperiod using white fluorescence neon lamps (Philips TL-D 90), at 30 μmol m^−2^ s^−1^ (low light). High light treatments were performed by irradiating the cells with 500 μmol m^−2^ s^−1^ for 5h, 2h after the onset of light, with the same light sources. Dark adaptation treatments were performed for 60h. Blue light (450nm, 1 μmol m^−2^ s^−1^) was applied for 10min, 30min, and 1h on dark-adapted cells in the absence and presence of 2 µM DCMU [3-(3,4-dichlorophenyl)-1,1-dimethylurea]. In the iron starvation experiments, *P. tricornutum* cells at an initial concentration of 2×10^5^ cells ml^–1^ were grown in f/2 artificial sea water medium (Allen *et al.*, 2008) modified to contain either 11 µM iron (iron-replete) or 5nM iron with the addition of 100 µM of the Fe^2+^ chelator FerroZine™ (iron-limited) (Stookey, 1970). Cells were harvested after 3 d to perform the analyses. Nitrogen starvation was achieved by diluting *P. tricornutum* cells to 2×10^5^ cells ml^–1^ in f/2 medium containing 1mM nitrate (NO_3_-replete) or 50 µM nitrate (NO_3_-limited). When cells attained a concentration of 1×10^6^ cells ml^–1^, they were re-diluted to 2×10^5^ cells ml^–1^ in their respective media and harvested after 3d, 2h after the onset of light, and then used for experiments.

### Generation of transgenic lines overexpressing the LHCX proteins

Vectors for LHCX overexpression were generated by cloning the full-length cDNA sequences of the four *LHCX* genes in the pKS-FcpBpAt-C-3HA vector (Siaut *et al.*, 2007), using the *Eco*RI and *Not*I restriction sites. The *LHCX* cDNAs were amplified by PCR using the primers described in Supplementary Table S1 at *JXB* online. Each vector was co-transformed with the pFCPFp-Shble vector for antibiotic selection into *P. tricornutum* Pt4 cells (DQ085804; De Martino *et al.*, 2007) by microparticle bombardment (Falciatore *et al.*, 1999). Transgenic lines were selected on 100 μg ml^–1^ phleomycin (Invitrogen) and screened by PCR using primers specific for the four LHCXs (Supplementary Table S1). Transgenic lines overexpressing the LHCX4 isoform in the Pt1 ecotype were also generated, as for the Pt4 ecotype.

### RNA extraction and qRT-PCR analysis

Total RNA was isolated from 10^8^ cells with TriPure isolation reagent (Roche Applied Science, IN, USA) according to the manufacturer’s instructions. Quantitative real-time PCR (qRT-PCR) was performed on wild-type cells and on the LHCX-overexpressing clones as described in De Riso *et al.* (2009). The relative quantification of the different *LHCX* transcripts was obtained using *RPS* (ribosomal protein small subunit 30S; ID10847) and *H4* (histone H4; ID34971) as reference genes, and by averaging of two reference genes using the geometric mean and the fold changes calculated with the 2^−ΔΔCt^ Livak method (Livak and Schmittgen, 2001). Primer sequences used in qRT-PCR analysis are reported in Supplementary Table S1.

### Protein extraction and western blot analysis

Western blot analyses were performed on total cell protein extracts prepared as in Bailleul *et al.* (2010), and resolved on 14% LDS–PAGE gels. Proteins were detected with different antibodies: anti-LHCSR (gift of G. Peers, University of California, Berkeley, CA, USA) (1:5000); anti-D2 (gift of J.-D. Rochaix, University of Geneva, Switzerland) (1:10 000); anti-PsaF (1:1000) and anti-βCF1 (1:10 000) (gift of F.-A. Wollman, Institut de Biologie Physico-Chimique, Paris, France); and anti-HA primary antibody (Roche) (1:2000). Proteins were revealed with Clarity reagents (Bio-Rad) and an Image Quant LAS4000 camera (GE Healthcare, USA).

### Chlorophyll fluorescence measurements

Light-induced fluorescence kinetics were measured using a fluorescence CCD camera recorder (JTS-10, BeamBio, France) as described (Johnson *et al.*, 2009) on cells at 1–2×10^6^ cells ml^–1^. *F*_v_/*F*_m_ was calculated as (*F*_m_–*F*_0_)/*F*_m_. NPQ was calculated as (*F*_m_–*F*_m_')/*F*_m_' (Bilger and Bjorkman, 1990), where *F*_m_ and *F*_m_' are the maximum fluorescence emission levels in the dark and light-acclimated cells, measured with a saturating pulse of light. All samples, except the 60h dark-adapted cells, were adapted to dim light (10 μmol m^−2^ s^−1^) for 15min at 18 °C before measurements. The maximal NPQ response was measured upon exposure for 10min to saturating green light of 950 μmol m^−2^ s^−1^. The relative electron transfer rate (rETR_PSII_) was measured with a JTS-10 spectrophotometer at different light intensities (20, 170, 260, 320, 520, and 950 µmol m^−2^ s^−1^), by changing light every 4min to minimize the photodamage. rETR_PSII_ was calculated as: Y2×light intensity, where Y2 is the efficiency of PSII.

### *In silico* analysis of the *LHCX* non-coding sequences

Determination of motif occurrence and *de novo* search of over-represented motifs in the 5'-flanking regions (1000bp or the entire intergenic sequence between the coding gene of interest and the upstream gene) and the introns of the Pt*LHCX* genes were performed by the use of the FIMO (v4.11.1) and MEME Suite (v4.9.1) tools (Bailey *et al.*, 2009), with a *p*-value cut-off of 0.0001. The Tomtom tool (v4.11.1) on the MEME Suite was used to compare motifs with known transcription binding sites. Microarray data from Alipanah *et al.* (2015) (accession GSE58946) were downloaded from the GEO database (http://www.ncbi.nlm.nih.gov/pubmed/23193258) using the GEO query package (http://www.ncbi.nlm.nih.gov/pubmed/17496320). Data were loaded and analysed in the R environment using the Limma package (http://www.ncbi.nlm.nih.gov/pubmed/25605792). Selection of transcripts categories: (i) up-regulated, log2 fold change (Fc) >3, adjusted *p*-value <0.01 (122 genes); (ii) down-regulated, Fc <–3, adjusted *p*-value <0.01 (200 genes). The *P. tricornutum* genome sequences and gff mapping of filtered gene models were downloaded from the JGI website and refer to Phatr2 (http://genome.jgi.doe.gov/Phatr2/Phatr2.home.html). The significance of motif enrichment was evaluated using the binomial test with a *p*-value cut-off of 0.05. All analyses were performed using custom scripts in Perl and R.

## Results

### LHCX family expansion in the diatom genomes

Several genes belonging to the LHCX family have already been identified in the genome of the pennate diatom *P. tricornutum* (Bailleul *et al.*, 2010) and the centric diatom *T. pseudonana* (Zhu and Green, 2010), the most established model species due to the availability of molecular toolkits for genetic manipulations (Apt *et al.*, 1996; Poulsen and Kroger, 2005; De Riso *et al.*, 2009; Trentacoste *et al.*, 2013; Daboussi *et al.*, 2014; Karas *et al.*, 2015). The recently available genome sequences of the pennate diatom *P. multiseries*, belonging to a widely distributed genus also comprising toxic species (Trainer *et al.*, 2012), and the centric diatom *T. oceanica*, a species adapted to oligotrophic conditions (Lommer *et al.*, 2012), opened up the possibility to extend this investigation to other ecologically relevant species. As summarized in Table 1, comparative analysis indicates an expansion of the *LHCX* family in diatoms, compared with the green algae: four members are present in *P. tricornutum*, five in *P. multiseries*, *T. pseudonana*, and *T. oceanica*, and up to 17 members have been found in the genome of the polar species *Fragilariopsis cylindrus* (B. Green and T. Mock, personal communication). Analysis of the intron–exon structure of all the available diatom *LHCX* genes revealed a variable number of introns (from zero to three) as well as variable intron and exon lengths (Table 1).

**Table 1.** List of the *LHCXs* identified in the diatom genomes

**Species**	**Name**	**ID**	**Chromosomal localization**	**Length (no. of amino acids**)	**No. of introns**
*Thalassiosira pseudonana*	LHCX1	264921	chr_23:365603–366232 (–)	209	0
	LHCX2	38879	chr_23:368273–368902 (+)	209	0
	LHCX4	270228	chr_5:1446306–1447125 (–)	231	0
	LHCX5	31128	chr_1:2849139–2850176 (–)	236	3
	LHCX6	12097	chr_23:366611–367378 (+)	255	0
*Phaeodactylum tricornutum*	LHCX1	27278	chr_7:996379–997300 (+)	206	1
	LHCX2	56312	chr_1:2471232–2472170 (+)	238	2
	LHCX3	44733	chr_5:76676–77606 (+)	206	1
	LHCX4	38720	chr_17:53010–53733 (+)	207	1
*Pseudo-nitzschia multiseries*	–	66239	scaffold_189:181982–182948 (+)	201	1
	–	238335	scaffold_95:121459–122306 (–)	202	1
	–	257821	scaffold_246:124909–125745 (+)	197	1
	–	264022	scaffold_1353:8720–9877 (+)	206	1
	–	283956	scaffold_38:284133–284828 (–)	231	0
*Thalassiosira oceanica*	–	Thaoc_09937	SuperContig To_g10869: 4.331–5.040 (–)	210	1
	–	Thaoc_12733	SuperContig To_g15184: 10.800–11.435 (–)	172	1
	–	Thaoc_28991	SuperContig To_g41561: 2.777–3.105 (+)	81	1
	–	Thaoc_31987	SuperContig To_g45669: 1–1.025 (–)	205	2
	–	Thaoc_32497	SuperContig To_g46152: 5.664–6.285 (–)	180	1

For *T. pseudonana* (Thaps3), *P. tricornutum* (Phatr2), and *P. multiseries* (Psemu1), ID numbers refer to the genome annotation in the JGI database (http://genome.jgi.doe.gov/). For *T. oceanica* (ThaOc_1.0), ID refers to the Ensembl Protist database (http://protists.ensembl.org/Thalassiosira_oceanica/Info/Index?db=core). (+) and (–) indicate the forward and reverse chromosomal or scaffolds, respectively. The protein length and intron numbers are also indicated.

### Light versus dark regulation of expression of the *LHCX* genes

**Fig. 1.**
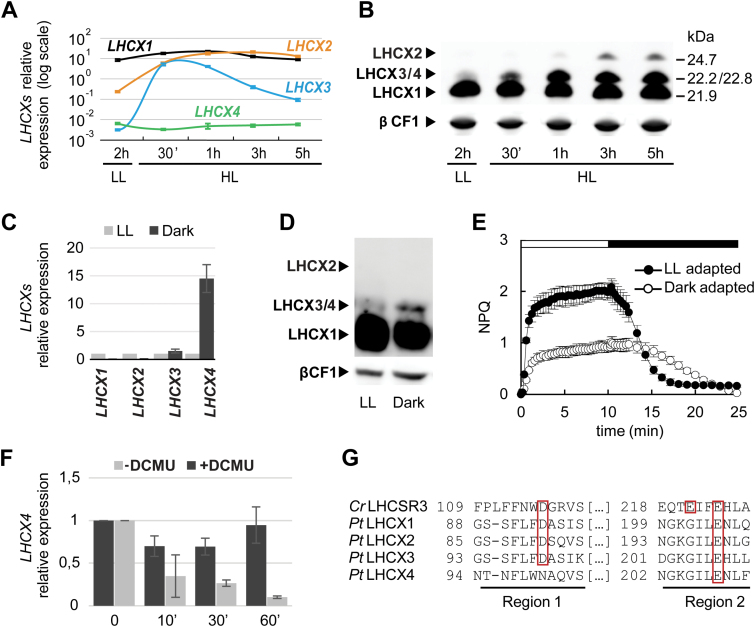
Light and dark regulation of *P. tricornutum* LHCXs. Analysis of the four *LHCX* transcripts by qRT-PCR (A) and of LHCX proteins (B) by western blotting in cells adapted to low light (LL) (12L/12D cycles), after exposure to LL for 2h then to high light (HL) for 30min, 1h, 3h, or 5h. mRNA levels were quantified by using *RPS* as the reference gene (A). Proteins were detected using the anti-LHCSR antibody which recognizes all the *Pt*LHCXs (arrowheads) and the anti-βCF1 antibody as loading control (B). Cells adapted to darkness for 60h were compared with those grown in LL for the analysis of LHCX transcripts (C), proteins (D), and NPQ (E). Relative transcript levels were determined using *RPS* as a reference, and values were normalized to gene expression levels in LL. LHCX proteins were detected as in (B). The horizontal bar in (E) indicates when the actinic light was on (white) or off (black). (F) *LHCX4* mRNAs in 60h dark-adapted cells (Time 0) and in response to 10min, 30min, or 1h of blue light (1 µmol m^−2^ s^−1^), in the presence (black) or absence (grey) of the inhibitor DCMU. Transcript levels were quantified by using *RPS* as the reference, and normalized to gene expression levels in the dark. Error bars represent ±SD of three technical replicates from one representative experiment in (A), and ±SD of three biological replicates in (C), (E), and (F). (G) Alignment of regions 1 and 2 of the *Chlamydomonas reinhardtii* LHCSR3 and *P. tricornutum* LHCX1, 2, 3, and 4 protein sequences. The boxes indicate the pH-sensing residues conserved between the LHCXs and LHCSR3. (This figure is available in colour at *JXB* online).

Independent gene expression studies performed in *P. tricornutum* cells suggest that the *LHCX* gene family is regulated by light via multiple regulatory pathways. To explore the mechanisms controlling the light responses of the *LHCX* genes further, we analysed mRNA and protein contents in cells exposed to different light conditions. We first monitored the expression of the *LHCX* genes in cells grown in low light (LL) and then exposed to high light (HL). In line with previous studies (Bailleul *et al.*, 2010; Lepetit *et al.*, 2013), qRT-PCR and western blot analyses (Fig. 1A and B, respectively) showed that LHCX1 is expressed at very high levels in LL-adapted cells, and that HL treatment slightly increases the LHCX1 content. Conversely, the isoforms 2 and 3 showed different responses to the LL to HL shift. The *LHCX2* transcripts, which are significantly less abundant than that of *LHCX1* in LL, rapidly increased following HL stress, reaching levels comparable with those of *LHCX1* after 1h HL exposure (Fig. 1A). This translated into an increase of the LHCX2 protein observed by western blot (Fig. 1B). However, the increase in the protein content was lower than that of the transcript, possibly because of a low affinity of the LHCSR antibody (Peers *et al.*, 2009) for the LHCX2 isoform. The *LHCX3* transcripts that were expressed at very low levels in LL quickly rose upon HL treatment, peaking after 30min and starting to decrease after 1h of light stress. Conversely, a different mRNA expression profile was found in the case of *LHCX4*, which, unlike the other isoforms, was barely detectable in both LL and HL conditions (Fig. 1A). The LHCX3 and LHCX4 proteins, having very similar molecular weights (22.8kDa and 22.2kDa, respectively), cannot be discriminated by western blot analysis. Based on the different transcriptional regulation of *LHCX3* and *LHCX4* by light, it is tempting to propose that the light-induced protein of ~22.8kDa reflects the accumulation of the LHCX3 isoform (Fig. 1B). However, in contrast to the transient induction of the *LHCX3* mRNAs, this protein is gradually accumulated during the LL to HL shift and it remains stable over the treatment. This discrepancy between transcript and protein expression profiles could be explained assuming that: (i) some post-transcriptional modifications regulate the accumulation of LHCX3 in the light; or (ii) the light-induced protein isoform at 22.8kDa also comprises the LHCX4 protein, which could be present in HL-exposed cells, along with LHCX3.

Previous reports (Lepetit *et al.*, 2013; Nymark *et al.*, 2013) indicate that the *LHCX4* transcript is induced in dark-adapted cells. Therefore, we extended the analysis of the expression of the four *LHCX* genes to cells adapted to prolonged darkness (60h). In these conditions, we observed a significant increase only of the *LHCX4* mRNAs (Fig. 1C). For the same reason as described above, we attributed the band of ~22kDa observed in the dark-adapted cells to the LHCX4 protein, although LHCX3 (Fig. 1D) could also be present. In the dark, cells were also showing a decreased NPQ capacity (Fig. 1E) and a slightly reduced PSII maximal quantum yield and overall photosynthetic electron flow capacity (Table 2). Other studies have revealed that blue light photoreceptors (Coesel *et al.*, 2009; Juhas *et al.*, 2014) and the redox state of the chloroplast (Lepetit *et al.*, 2013) could both contribute to the light regulation of *LHCX1*, *2*, and *3* gene expression. Thus, we tested the possible role of these processes in the inhibition of *LHCX4* expression upon light exposure. We irradiated dark-adapted cells with low intensity blue light (1 µmol m^−2^ s^−1^) during 1h, in the presence or absence of the PSII inhibitor DCMU (Fig. 1F). The analysis revealed that the *LHCX4* expression is repressed even at such low light irradiance. Moreover, this repression is lost by poisoning photosynthesis with DCMU, suggesting that this process plays an active role in the light-induced repression of *LHCX4*.

**Table 2.** Photosynthetic parameters of the *P. tricornutum* wild type and transgenic lines

**Strain**	**Conditions**	***F***_**v**_ **/*F***_**m**_	**rETR**_**PSII**_	**NPQ max**
Pt1	LL	0.66±0.03	74.4±1.2	2.1±0.1
Pt1	Dark	0.60±0.02	67.2±4.0	1.0±0.1
Pt1	+Fe	0.65±0.003	72.6±4.8	2.2±0.3
Pt1	–Fe	0.20±0.004	25.7±1.8	4.4±0.3
Pt1	+N	0.65±0.006	74.4±2.4	2.1±0.1
Pt1	–N	0.40±0.008	27.5±3.2	3.2±0.4
Pt4	WT	0.68±0.01	79.3±2.6	0.83±0.04
Pt4	EVL	0.66±0.01	72.5±3.5	0.82±0.03
Pt4	OE1	0.67±0.01	70.7±2.6	1.00±0.1
Pt4	OE2.5	0.67±0.01	70.0±1.6	1.06±0.05
Pt4	OE2.20	0.66±0.01	69.3±1.6	1.02±0.08
Pt4	OE3.12	0.66±0.01	75.8±4.9	1.04±0.07
Pt4	OE3.33	0.68±0.03	71.0±3.1	1.00±0.1
Pt4	OE4.11	**0.59±0.01**	68.7±1.9	1.03±0.01
Pt4	OE4.13	**0.58±0.02**	69.4±1.3	1.07±0.04

PSII efficiency (*F*_v_/*F*_m_) and relative electron transport rate (rETR_PSII_) in different growth conditions are reported. rETR_PSII_ was measured at 260 µmol photons m^−2^ s^−1^ light intensity and calculated as: rETR_PSII_=ɸPSII×actinic light intensity. Non-photochemical quenching (NPQ) was measured with an actinic light intensity of 950 µmol photons m^−2^ s^−1^ and calculated as in Maxwell and Johnson (2000). Data are the average of three biological replicates ±SD.

A recent study in *Chlamydomonas reinhardtii* showed that the activity of the protein LHCSR3 is regulated by the reversible protonation of three specific amino acidic residues following luminal pH acidification in the light (Ballottari *et al.*, 2016). In order to assess if this mechanism is conserved in diatoms, we analysed the *P. tricornutum* LHCX protein sequences. We found that LHCX1, 2, and 3 possess two of the three amino acids identified in LHCSR3 in conserved positions (Fig. 1G; Supplementary Fig. S2), suggesting that the pH-triggered activation of qE could be conserved in diatoms. On the other hand, only one of these protonatable residues was found in LHCX4.

### LHCX expression in iron starvation

**Fig. 2.**
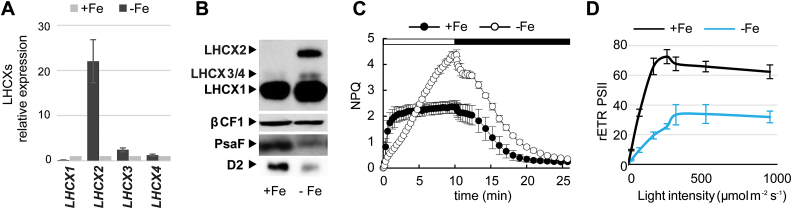
Effect of iron starvation on *P. tricornutum* LHCX expression and photophysiology. Experiments were performed on cells grown in iron-replete (11 µM, +Fe) or iron-limited (5nM iron+100 µM FerroZine™, –Fe) conditions: (A) qRT-PCR analysis of *LHCX* transcripts in –Fe, normalized against the +Fe condition and using *RPS* and *H4* as reference genes. (B) Immunoblot analysis of the LHCX, D2, and PsaF proteins, using βCF1 as loading control. NPQ capacity (C) and relative electron transfer rates (rETR_PSII_) (D) of cells grown in +Fe or –Fe. The horizontal bar in (C) indicates when the actinic light was on (white) or off (black). rETR_PSII_ was measured at different light intensities (20, 170, 260, 320, 520, and 950 µmol m^−2^ s^−1^). In (A), (C), and (D), error bars represent ±SD of three biological replicates. (This figure is available in colour at *JXB* online).

Besides light, nutrient availability also affects chloroplast activity (Wilhelm *et al.*, 2006; Gross, 2012). In many oceanic regions, iron is a major limiting factor for diatom distribution. A general down-regulation of photosynthesis has been reported in iron starvation in several diatom species (Laroche *et al.*, 1995; Allen *et al.*, 2008; Hohner *et al.*, 2013), with a consequent decrease of the carbon fixation reactions, growth rate, and cell size. Since increased NPQ was previously observed in iron-starved *P. tricornutum* cells (Allen *et al.*, 2008), we compared the expression of the different LHCX isoforms in cells grown under Fe-replete and Fe-limited conditions. We found that while a slight induction of the other LHCX proteins was seen, the *LHCX2* transcript was greatly induced in iron-limited cells (Fig. 2A), leading to a significant accumulation of the LHCX2 protein (Fig. 2B). Fe limitation also enhanced NPQ, while slowing down its kinetics (Fig. 2C), possibly because of a slower diadinoxanthin de-epoxidation rate. We also observed a severe impairment of the photosynthetic capacity in iron limitation as indicated by the decrease in *F*_v_/*F*_m_ (Table 2) and in the PSII maximal electron transport rate (rETR_PSII_) (Fig. 2D; see also Allen *et al.*, 2008). The decreased maximal rETR_PSII_ was probably caused by a diminished capacity for carbon fixation. Moreover, in agreement with previous studies (Allen *et al.*, 2008; Thamatrakoln *et al.*, 2013), we observed a decrease in the amount of PSI (PsaF), which is the complex with the highest Fe content. This complex has been already shown to represent the first target of Fe limitation (Moseley *et al.*, 2002). We also found a significant decrease in PSII (D2 protein), which was probably degraded because of sustained photoinhibition (see also Allen *et al.*, 2008) (Fig. 2B).

### LHCX expression in nitrogen starvation

**Fig. 3.**
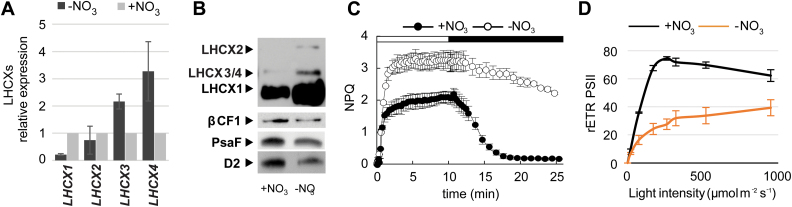
Effect of nitrogen starvation on *P. tricornutum* LHCX expression and photophysiology. Experiments were performed on cells grown in nitrogen-replete (1mM, +NO_3_^–^) or nitrogen starvation (50 µM, –NO_3_^–^) conditions: (A) qRT-PCR analysis of *LHCX* transcripts in –NO_3_^–^, normalized against the values in the +NO_3_^–^ condition, and using *RPS* and *H4* as reference genes. (B) Immunoblot analysis of the LHCX, D2, and PsaF proteins, using βCF1 as loading control. NPQ capacity (C) and relative electron transfer rates (rETR_PSII_) (D) of cells grown in +NO_3_^–^ and –NO_3_^–^ conditions. The horizontal bar in (C) indicates when the actinic light was on (white) or off (black). rETR_PSII_ was measured at different light intensities (20, 170, 260, 320, 520, and 950 µmol m^−2^ s^−1^). In (A), (C), and (D), error bars represent ±SD of three biological replicates. (This figure is available in colour at *JXB* online).

Besides iron, nitrogen (N) is also a limiting resource for diatoms (Mills *et al.*, 2008; Moore *et al.*, 2013; Rogato *et al.*, 2015). Recent transcriptomic and proteomic analysis highlighted important metabolic modifications under N starvation, such as the up-regulation of nitrogen assimilation enzymes, the recycling of intracellular nitrogen-containing compounds from the photosynthetic apparatus and other sources, and the increase in lipid content as a consequence of remodelling of intermediate metabolism (Allen *et al.*, 2011; Palmucci *et al.*, 2011; Hockin *et al.*, 2012; Alipanah *et al.*, 2015; Levitan *et al.*, 2015; Matthijs *et al.*, 2016). We found that N limitation also has a significant effect on the expression of the LHCXs. In particular, N limitation triggered the induction of *LHCX3* and *LHCX4* mRNAs (Fig. 3A) and of LHCX3/4 proteins (Fig. 3B). The increase of the LHCX1 and 2 isoforms was only visible at the protein level (Fig. 3B). Up-regulation of the LHCX proteins in N limitation correlated with an increase of the NPQ capacity (Fig. 3C). We note that NPQ was slowly relaxing upon dark exposure of N-limited cells, possibly reflecting the repression of the genes encoding the xanthophyll cycle enzymes including the zeaxanthin epoxidase (see Supplementary Fig. S3), when analysing available microarray data from N-depleted cells (Alipanah *et al.*, 2015). The N limitation also led to a drastically reduced *F*_v_/*F*_m_ (Table 2) and a lower maximal rETR_PSII_ (Fig. 3D). We also detected a reduced content of PSII and PSI proteins (Fig. 3B), in line with previous omic studies pointing to a general decrease of the photosynthetic capacity.

### Analysis of the *LHCX* non-coding regions

Due to the observed transcriptional responses of *LHCX* genes in different light and nutrient conditions, we searched for known and potentially novel regulatory motifs in the 5'-flanking regions and the intronic sequences of the four isoforms (see Table 3; Supplementary Fig. S1). Because of their involvement in integrating light signals with CO_2_/cAMP-induced transcriptional responses, we searched for the three CO_2_/cAMP-responsive *cis*-regulatory elements (CCREs) identified in Ohno *et al.* (2012) and further characterized in Tanaka *et al.* (2016). Interestingly, we found CCRE-1 in the 5'-flanking sequences of *LHCX1* and *4*, CCRE-2 in *1*, *2*, and *4*, and CCRE-3 in *LHCX4*. These *cis*-regulatory elements may participate in the light-mediated regulation of the four *LHCX* genes.

**Table 3.** The identified regulatory motifs and their occurrence in the *P. tricornutum LHCX* non-coding sequences

	**Sequence**	***LHCX1***	***LHCX2***	***LHCX3***	***LHCX4***
MOTIF 1	TCA[CT][AT]GTCA	2	2	1	–
MOTIF 2	CGAACCTTGG	–	–	2	–
MOTIF 3	CCT[GC]TCCGTA	–	–	2	–
MOTIF 4	GAGTCCATCG	–	–	–	2
MOTIF 5	CGATCACGGC	–	–	–	2
MOTIF 6	[TA]TGACTG	–	1	1	1
CCRE-1	TGACGT	1	–	–	1
CCRE-2	ACGTCA	1	1	–	1
CCRE-3	TGACGC	–	–	–	1

In contrast, the two *P. tricornutum* iron-responsive elements identified in Yoshinaga *et al.* (2014) are not present in the analysed non-coding regions, suggesting that a different transcription factor should be involved in modulating the *LHCX2* transcriptional response to iron availability. Similarly, we could not find the two *P. tricornutum* motifs identified as responsive to short-term nitrogen deprivation (from 4h to 20h) in Matthijs *et al.* (2016), suggesting that distinct regulatory circuits may act in the short- and long-term acclimation to nitrogen deprivation.

To pinpoint possible novel regulatory motifs, we also scanned the non-coding sequences of the four isoforms using the MEME Suite program (Bailey *et al.*, 2009). The analysis revealed six motifs repeated at least twice in each isoform and/or shared by more than one isoform (Table 3; Supplementary Fig. S1). None of the identified motifs corresponds to a known transcription factor-binding site. This suggests that these motifs could represent novel diatom-specific *cis*-regulatory elements. In order to examine the potential involvement of the identified motifs in the long-term nitrate deprivation transcriptional responses of *LHCX* genes, we analysed a published microarray data set performed on 48h and 72h nitrogen-deprived *P. tricornutum* cells (Alipanah *et al.*, 2015). We compared the frequency of the six identified motifs in the 5'-flanking sequences of responsive and unresponsive transcripts. Interestingly, motif 6 ([T-A]TGACTG) was significantly enriched (*p*=0.035) in the 5'-flanking sequences of genes up-regulated in response to nitrogen starvation compared with down-regulated genes. The result suggests that motif 6 may be involved in gene transcriptional regulation in cells exposed to prolonged nitrogen starvation.

### Modulation of *LHCX* gene expression in *P. tricornutum* transgenic lines

**Fig. 4.**
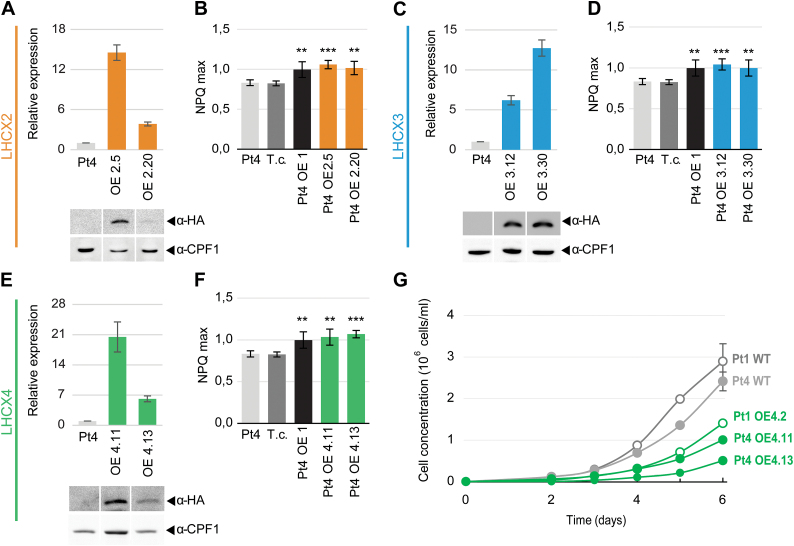
*Phaeodactylum tricornutum* Pt4 ecotype lines overexpressing the *LHCX* genes. (A), (C), (E) LHCX transcript (upper panels) and protein (lower panels) analyses in the Pt4 wild type and transgenic strains overexpressing HA-tagged *LHCX2* (A), *LHCX3* (C), or *LHCX4* (E). Transcript abundance was measured by qRT-PCR using *RPS* as the reference gene and normalized to the wild type expression value. Tagged proteins were detected by immunoblot using an anti-HA antibody, and an anti-CPF1 antibody as loading control. Bands are taken from the same blots but from non-adjacent lanes. (B), (D), (F) NPQ max capacity in the Pt4 wild type, in a transgenic strain expressing the vector for antibiotic resistance (transformation control, T.c.), and in independent transgenic lines overexpressing *LHCX1* (OE1), *LHCX2* (OE2), *LHCX3* (OE3), and *LHCX4* (OE4) genes. Asterisks indicate the results of two-tailed Student *t*-tests: **p<0.01; ***p<0.001. (G) Growth curves of Pt4 and Pt1 wild-type strains and Pt4 and Pt1 transgenic lines overexpressing the *LHCX4* (OE4) gene, grown in 12L/12D cycles (50 µmol m^−2^ s^−1^). In all the experiments, *n*≥3, and bars represent ±SD. (This figure is available in colour at *JXB* online).

A role in the regulation of the NPQ in *P. tricornutum* has been proven for the LHCX1 protein by characterizing transgenic lines with a modulated content of LHCX1 by either gene silencing or gene overexpression (Bailleul *et al.*, 2010). Unfortunately, all the attempts to down-regulate the expression of *LHCX2*, *3*, or *4* have been unsuccessful. Therefore, to explore their function, we opted for the strategy used in Bailleul *et al.* (2010), and tried to rescue the intrinsically lower NPQ capacity of the Pt4 ecotype. To this end, independent transgenic Pt4 lines were generated, bearing a vector in which the *LHCX2*, *3*, or *4* genes were expressed under the control of the *P. tricornutum FCPB (LHCF2*) promoter. A HA-tag was fused to the C-terminal end of the *LHCX* transgenes to allow the specific detection of the transgenic proteins. qRT-PCR and western blot analyses (Fig. 4A, C, E) on independent transgenic lines confirmed the expression of the transgenic LHCX isoforms. NPQ analyses (Fig. 4B, D, F) showed that the overexpression of each LHCX isoform generated a modest, but statistically significant, increase in the NPQ capacity compared with the Pt4 wild type as well as compared with a transgenic line transformed only with the antibiotic resistance gene and used as control. Strikingly, we found that all the transgenic lines showed a similar NPQ increase, regardless of which isoform was overexpressed and the different overexpression levels.

We also checked the possible effect of LHCX overexpression on growth and photosynthetic capacity. For the lines overexpressing the LHCX2 and LHCX3 proteins, we did not observe any altered phenotype (Table 2). In contrast, the Pt4 lines overexpressing LHCX4 showed a reduced PSII efficiency (Table 2). By performing a growth curve analysis, we also observed that these overexpressing lines showed a lag phase lasting 2–3 d (Fig. 4G), which was not the case in wild-type cells. A similar effect on growth was also observed in transgenic lines in which the *LHCX4* gene was overexpressed in the Pt1 ecotype (Fig. 4G).

## Discussion

**Fig. 5.**
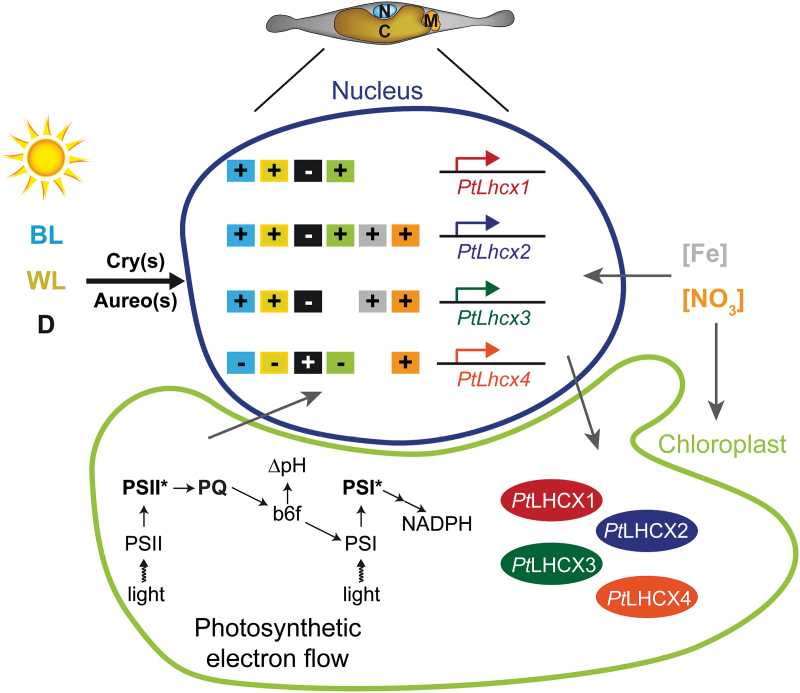
Model of the *P. tricornutum* LHCX regulation. Scheme summarizing the multiple external signals and stresses that differentially regulate the expression of the four LHCXs. The *LHCX* genes are shown in the nucleus and the LHCX proteins in the chloroplast. + and – boxes indicate positive and negative transcriptional regulation, respectively, in response to white light (yellow), blue light (blue, through the cryptochromes, Cry, and aureochromes, Aureo, photoreceptors), darkness (black), chloroplast signals (green), iron starvation (grey), and nitrogen starvation (orange). In the *P. tricornutum* cell: N, nucleus; C, chloroplast; M, mitochondrion. In the chloroplast: PSI and PSII, photosystem I and II, respectively; PSI* and PSII*, excited photosystems; PQ, plastoquinone pool; b_6_f, cytochrome *b*_6_ *f* complex; ΔpH, proton gradient; NADPH, redox potential.

The presence of multiple *LHCX* genes in all the diatom genomes analysed to date strongly suggests that the expansion of this gene family is a common feature of these algae and may represent an adaptive trait to cope with highly variable environmental conditions. To investigate this scenario, in this work we correlated LHCX expression profiles with the photosynthetic and photoprotective performances in variable experimental conditions, including changes in light irradiance and nutrient availability. These analyses revealed that the four *P. tricornutum LHCX* genes respond differently to various environmental cues, as summarized in Fig. 5.

The analyses of the mRNA and protein responses indicate that amounts of the different LHCXs are tightly regulated at the transcriptional, and probably also the post-translational level. As LHCX3 and LHCX4 have a similar size, it was not possible to quantify the amount of these two proteins under the different stresses using one-dimensional electrophoresis. However, considering the transcript and biochemical analyses together (in the case of LHCX2 and LHCX1), it seems that LHCX1 is always expressed at high levels even in non-stress conditions, which is consistent with it having a pivotal role in NPQ regulation and light acclimation as proposed previously (Bailleul *et al.*, 2010).

LHCX2 and 3 are induced following high light stress, where they may contribute to increase the diatom photoprotection capacity. Their induction, as well as the accumulation of LHCX1, may result from the integration of different signals. Two members of the blue light-sensing cryptochrome photolyase family, CPF1 (Coesel *et al.*, 2009) and CRYP (Juhas *et al.*, 2014), modulate the light-dependent expression of LHCX1, LHCX2, and LHCX3. Also, the recently identified Aureochrome 1a blue light photoreceptor, which regulates *P. tricornutum* photoacclimation (Schellenberger Costa *et al.*, 2013), may affect how much of each LHCX there is in a cell. Moreover, chloroplast activity, through the redox state of the plastoquinone pool, may also regulate *LHCX1* and *LHCX2* gene expression in HL (Lepetit *et al.*, 2013).

A different regulation pattern is seen in the case of LHCX4, the only isoform which is induced in the absence of light. The amount of *LHCX4* mRNA rapidly decreases following a dark to light transition, and this repression is lost when photosynthesis is halted with the PSII inhibitor DCMU. This suggests that chloroplast-derived signals could participate in inhibiting gene expression, even at very low light irradiance, by an as yet unknown process. The peculiar trend observed in the LHCX4 light response suggests a possible role for this protein in *P. tricornutum* photoacclimation. The increased LHCX4 transcript and possibly protein content is mirrored by a decrease in NPQ capacity and a slightly reduced *F*_v_/*F*_m_ in the dark-adapted cells, compared with cells grown in the light (Fig. 1E; Table 2). Moreover, reduced PSII efficiency and slightly altered growth were observed in cells overexpressing LHCX4 in the light (Fig. 4G), suggesting that LHCX4 could have a negative impact on chloroplast physiology. Indeed, a comparative analysis of the *P. tricornutum* LHCX protein sequences indicates that LHCX4 lacks key protonatable residues that in *Chlamydomonas* are involved in NPQ onset when the lumen acidifies (Ballottari *et al.*, 2016). These residues are, however, conserved in the LHCX1, 2, and 3 isoforms. According to the model established in green algae for the protein LHCSR3, these residues diminish their electrostatic repulsion upon protonation, allowing a rearrangement of the protein structure and pigment orientation and enhancement of the quenching capacity (Ballottari *et al.*, 2016). The substitution in LHCX4 of the acidic residues (aspartate and glutamate) with non-protonatable residues (asparagine and glycine) would prevent such regulation. Instead, LHCX4 could contribute to the observed capacity of *P. tricornutum* to survive long periods in the dark and its repression could be needed for a rapid acclimation following re-illumination (Nymark at al., 2013). Consistent with this, high *LHCX* gene expression has also been observed in sea-ice algal communities dominated by diatoms that have adapted to the polar night (Pearson *et al.*, 2015).

Besides the light and redox signals discussed above, our study also shows that differences in the availability of iron and nitrogen strongly affect the expression of the different LHCXs. The signalling cascades controlling these responses are still largely unknown, but they probably involve multiple regulatory pathways into the nucleus and chloroplast, considering that these nutrients are essential for diatom photosynthesis and growth (Table 2; Fig. 5). Nitrogen starvation induces a general increase of all the LHCX isoforms, including LHCX4 that is normally repressed in light-grown cells (Fig. 3). We can hypothesize that the general increase of the LHCX content is needed to protect the photosynthetic apparatus, which is strongly affected by nitrogen deprivation, as shown by the drastically reduced *F*_v_/*F*_m_ (Table 2), the lower maximal rETR_PSII_ (Fig. 3D), and the reduction of PSI and PSII protein content. Interestingly, an opposite trend is observed for the main enzymes of the xanthophyll cycle, which are either not induced or are repressed in cells grown in similar nitrogen stress conditions (Supplementary Fig. S3). Thus, in nitrogen starvation, the LHCXs could represent the major contributors to the observed NPQ increase (Fig. 3C).

At variance with nitrogen starvation, iron starvation has a more specific effect on LHCX expression. A strong induction of the LHCX2 mRNA and protein levels (Fig. 2) compared with the other isoforms was observed, pinpointing this isoform as the most likely regulator of the increased NPQ capacity observed in iron stress (Fig. 2C). NPQ in iron-limiting conditions is characterized by a slow induction and a complete relaxation in the dark. These slow induction kinetics might reflect either lower concentrations of the pH-activated de-epoxidase enzyme or its cofactor ascorbate (Grouneva *et al.*, 2006) or slower acidification of the thylakoid lumen due to a reduced photosynthetic activity. Indeed, the photosynthetic capacity is severely impaired when iron is limiting, as demonstrated by the reduction in PSI and PSII subunits (Fig. 2B), but also the lower *F*_v_/*F*_m_ (Table 2) and rETR_PSII_ (Fig. 2D). The decreased electron flow per PSII could also reflect a decrease in the iron-containing cytochrome *b*_6_ *f* complex, as previously shown for other iron-limited diatoms (Strzepek and Harrison, 2004; Thamatrakoln *et al.*, 2013).

The observations made in this and in previous studies about the complex LHCX regulation in response to different signals prompted us to explore their possible functions in *P. tricornutum*, by modulating their expression in a natural Pt4 strain characterized by constitutive lower NPQ levels (Fig. 4). We observed that the increased expression of all the tested isoforms generates a small but still consistent increase in the NPQ levels, suggesting a potential involvement of the diverse proteins in NPQ modulation, as previously shown for LHCX1 (Bailleul *et al.*, 2010). However, we also noticed that different overexpressing lines with different transcript and protein levels showed a similar NPQ increase. It is difficult to interpret these first results, especially in the case of lines overexpressing LHCX4, whose endogenous expression is inhibited by light (Fig. 1F). They probably reflect the complexity of NPQ regulation in diatoms, where the presence of multiple players (e.g. several LHCXs and enzymes of the xanthophyll cycle) possibly tend to reduce the consequences on NPQ of genetic modifications of the qE machinery.

Finally, the exploration of the 5'-flanking regions and intronic sequences of the *LHCX* genes revealed the presence of known and potentially novel *cis*-regulatory elements that may contribute to the transcriptional regulation of the different isoforms in stress conditions. We revealed an uneven distribution of the CCREs (Ohno *et al.*, 2012; Tanaka *et al.*, 2016) in the four *LHCX* genes that may be linked to their different light-mediated transcriptional responses. In addition, we identified a 7bp motif in the non-coding sequences of *LHCX2*, *3*, and *4*. Using genome-wide transcriptomic data, we found this motif specifically enriched in long-term nitrogen starvation-induced genes, suggesting a possible involvement in the regulation of gene expression in response to nitrogen fluctuations. Although additional studies are required to demonstrate the functionality of these motifs, their discovery may represent a starting point for the identification of the *LHCX* regulators in the diatom acclimation mechanisms to stress.

### Outlook

Here we discovered that the four *P. tricornutum* LHCXs are regulated in a sophisticated way (Fig. 5). Different and probably interconnected regulatory pathways activated by different signals and stresses tightly control the amount of each LHCX isoform in the cell. By narrowing down the specific growth conditions in which the different LHCXs are required, our results set the basis for future work to define the function of each isoform in the regulation of chloroplast physiology. The generation of new transgenic lines in which the content of each LHCX isoform is specifically modulated will be instrumental in assessing whether they act with the NPQ regulator LHCX1, or play other specific roles. Considering the robustness of LHCX1 expression in all the conditions tested, future studies will probably require the use of new LHCX1 loss-of-function diatom strains. Additional information about the association of LHCXs with photosynthetic complexes and pigments will also be necessary to understand the role played by the expanded *LHCX* gene family in the efficient acclimation of diatoms to environmental changes.

## Supplementary data

Supplementary data are available at *JXB* online.

Figure S1. Localization of the enriched motifs in non-coding regions of *P. tricornutum LHCX* genes.

Figure S2. Alignment of the LHCX proteins and three-dimensional model of LHCX1.

Figure S3. Expression of the *P. tricornutum* xanthophyll cycle genes in nitrogen starvation.

Table S1. List of the oligonucleotides used in this work.

Supplementary Data
