# Supplementary material for: Multisignal control of expression of the LHCX protein family in the marine diatom Phaeodactylum tricornutum
Source: J Exp Bot. 2016 May 25;67(13):3939–51. doi: 10.1093/jxb/erw198 (PMC4915529; doi:10.1093/jxb/erw198)
Supplement: Supplementary Data [file supp_67_13_3939__index.html]

Multisignal control of expression of the LHCX protein family in the marine diatom Phaeodactylum tricornutum — Multisignal control of expression of the LHCX protein family in the marine diatom Phaeodactylum tricornutum — Supplementary Data 

# Multisignal control of expression of the LHCX protein family in the marine diatom *Phaeodactylum tricornutum*

## Supplementary Data

Data files

- supplementary\_figure\_S1\_S3\_Table\_S1.pdf - Supplementary Data
