# Supplementary material for: Multisignal control of expression of the LHCX protein family in the marine diatom Phaeodactylum tricornutum
Source: J Exp Bot. 2016 May 25;67(13):3939–51. doi: 10.1093/jxb/erw198 (PMC4915529; doi:10.1093/jxb/erw198)
Supplement: Supplementary Data [file supp_erw198_supplementary_figure_S1_S3_Table_S1.pdf]

Supplementary Data

Multi-signal control of the expression of the LHCX protein family in the marine diatom *Phaeodactylum tricornutum*

Lucilla Taddei[,#], Giulio Rocco Stella[#], Alessandra Rogato[#], Benjamin Bailleul, Antonio Emidio Fortunato, Rossella Annunziata, Remo Sanges, Michael Thaler, Bernard Lepetit, Johann Lavaud, Marianne Jaubert, Giovanni Finazzi, Jean-Pierre Bouly, Angela Falciatore

PtLhcx1

5'-flanking sequence

TCAAAGGAAGGAATGTAGAGTAAGCAAACCATCATTTCGCCGCTTCCATTACCAAGAGAACCGACCAAGAGCCCTACCTTGTATGTCATTCGTATAAAC
GTGCACTGAATCGCCCCAGCATGTGAACGCACCATCTCGAATGGCCATCGACAATTCCTTCCGACCGAAAACGCAAACACCACAAGCTTTCTGAGAGGG
CTTATTCTCACATCCGTACATGACATCTTCGTTGAAGTCTACTAGGACAAGAAGCCACCGAGTCTTGTGCCGAACCATCGTGTAGCAAATCGGCGAGGC
GAAGCTATTACAACCTTAACTGGGTCAGTACAGCGTGTTGATTGTGAACAAAACCAATGGCCAGACGGATTCGCAAGTTCACGACAGCTTTCGACGGTG
AGAATGATGGTATCGCTTCGGGTCGGGTCGGTACTTTGCGGGACTGGCAACGTCGGCAGAGGGACTCACGGTCACTTCACTGTCTGTATCGACTGACGA
CTGACTCGTCTGATTCACTGTCAGGTTTCTGATCTAAGTTTATCAAATGCTACTACTTGAGGTAGTCGTATCATTTGAAACTGCAACTTACAAAATCGA
AGGTCCTTGCAACATTTCTGACATGGGTAATTTCATAGGATGTTACAATGTCGAGCTTGTATCACTCGGATACAACAGACCACTTTAGGAAAGGGATTT
TTTTTTGTCGAAAATAGGATGACATCAACAGGAAGTACGTTCATAGCCCTTTATTTATAATTGGATGGGTCAATCCGACGTACGCATGTGTCTTTTGAC
CTTGTCATCCATAACCAGATTGACCCGTCACTGTGCAATACGTCACGCCAATGAAAGACCCCCGGATAAAGGGCCTAAAATTCAGTCTCGTGCAAAACA
CGCAGGATGATGCACCTGAACAGGTGACGTGTTCGCGCAGTCGAATGAGTTCCGAAGCCATCAGAGGCGAATTTATTTTGCAACTGTTTGACCCCATAA
AACCATTCCT

PtLhcx2

5'-flanking sequence

ATCACGTGAGATATCACTGATATCTCACGTCAATAGACAAGCTGCTTTACAATGGGTGAAAACAACCACCACACGAAACAAGTTCCTGTAACTCTAAAT
ATTGATTGTAATTGGTGAAGAAAGCATTTTCCTGACTGTGGGTACATGGAATACATCCTTTTTTGATAAATGTACAAACAAAGACTCATTTACTGTTAG
TCATAGTCAAGCGCAGTGCGTACTTGACTGTGAAAAAATCGAGCTCTGAAAGGGTTAAAGTTCAAGAGGTACAAGGTAGCTGGTTTGCGTGTGTCAGGG
CATGGCCTACCTGACTCATGCATTCCGCGTGCCACAAAAACTCACAGTCAGAGAGCCACTCCGAGAATCCTCCAGAATTTCGTGGAAGATTTTTTCGTC
ATCTTTTTCCAGTTCTCCGCATAGCTCTCATAGTTTCGTTTGCGTTCTTCGTCAACACACCGCAAAAAGATATACGTCCAATTCCAGCCCACTACGTAC
ACC

PtLhcx3

5'-flanking sequence

AGAAATGTCAAGATCATGGATTGGTTTCAAGGACACATGACATTGTAATCCTTACGGCTATTTCGATCCGAACCCAGAGTATGGTAGGTAGGTAGTGAC
ACGAGCGAGTCATAGAATTGTTGTTTGATCAGACAATCTGTACACTTATTCGACCATGTCTATTTATTACCACACGTGTTTGTACGCGCCAAACAGATT
CTTTCGCTAGAATCCGACTGATCGACGACCACACAGAGAGAGGGACTGACGAAGTACAACCACGCGACGAAACCACCACGGCAACACAACATAGCGTTA
ATTTTACAATTAGTGCTGACTAACAGCGTATACTGTCAACAGAGTCCGATGACTGGCTGACAATGACTTGGAACGCCAATTTGCATTGATAGTGACTAT
GAGTTCCAAGCTTCGATCGGGCCGAACCAAGTAGAAAGATACATGTAAACGACTACCTATATACTGGCTGATTCAATCCGACCAGCAGAGTAACCCCAT
CTGGTGTCGATTCCTCTGCGAATACTCTAGAGGTATCACGGTATGTATATAGTATATAGTACTCGTCGCCCACGGTTGTCTTCGATTCCCATCCATCCT
GTTACGGACAGGAACCAGACACGACACGGCCCTACCTCTCCGTATCCGATCCTTGCCATCACTTTTGGGGTACTTTCGAAAAAGACTGATTCATAGTCA
AATGCCTTCTGACGGATCCAGAGGGTACGCAGAGTTCTGCTGACGAACTGTCTCGTACACTCATTCTTGGTTGTGGAAATCATCTCCCTACACACTACG
CTCCAAAATGATAACTCTGCACAATTCACACAATATCCTAAACAGTACACTTGCGATCGCACTTGCAACCCACGTACCACCACCAGTACCAAACACAAT
CATTACACC

intron1

GTACGTCTACGCGAACCTTGGCATTTTCCAGGTACCGCAGTGTCGGTGTGTTACAGTTAGTGTCGTCCGCGATTGCATTCCTCACGAACCACACTCGTT
CCGCTTTGTCCCGGCAG

PtLhcx4

5'-flanking sequence

GAAAACTCTAGCCCGGCACGGCAGGCCGTCCTCTGCACCTGTAGTGAAGCGAATGTCGAATGTCCATGTCGAAGTCGTAGGAGACGCCGACAGAGAAAC
CACGGCTATCATGTAAACAATATGCAAGAGTACCTTACTTATCGAACATGCTGGAGTTATTTGCTGTTGATTTAAGTATAATTTAATTGACGCAGTATA
TATAAATCAGTGCACATAAAGTTTGACGAAACCTCATAGTTCGAGTTTTGTTATGCATCACTGAATTTCCAGAACATGGCAAATCCTGTAACACCTATT
TTTACACTTACATCGATAATTGGTTACAATCAAGCCTTGGCCCACAAGCACCAGCTAACATAAGTGGTCACAGCAATTTTAGTGGTTCGTGTCATACTC
AAGCGACGAATCACCCAATTCTCGGAAGTAAACCGAACTCGATCACGGCGATGCAAGTCCTTTAATTCTCACGAGTCCATCGAACTCGATCACGGCGAT
GCAAGTCCTTTTCTCAGGAGTCCATCGAGCTCGATAACAACGAAGCAGGTCGACGCCACGTGTTCCAGAAATTGGTTGCCTTCTGTCGACTAATCGTTC
TTTCGATCTCGAATGAATTCCTTTTGCTTTCCATACTTGTTGACGTCGTCGAGGGGAGTCTGTGAGAGCAGGCAGCGAGTCGTTACTCCTCGGATTTGT
TCATTCCCAGTCATCGAAGCAGTGGTCGTTTGACTCTCACCTTACAATTTGAGATCCTTGCGGTTTTCGAGAGAGTGAGACACGTCAAACGCACTCAGC
CAGT

Motif 1: TCA[CT][AT]GTCA
Motif 2: CGAACCTTGG
Motif 3: CCT[GC]TCCGTA
Motif 4: GAGTCCATCG
Motif 5: CGATCACGGC
Motif 6: [TA]TGACTG
CCRE 1: TGACGT
CCRE 2: ACGTCA
CCRE 3: TGACGC

Fig. S1: Localization of the enriched motifs in *LHCXs* non-coding regions.

A

```
CrLHCSR3     ------MLANVVSRKASGLRQT-PARATVAVKSVSGRR---TTAAEPQTAAPVAAEDVFA  50
PtLHCX1      MKFAATILALI-G-SAAAFAPA-------------------------QTSRA--STSLQY  31
PtLHCX2      MKLSLAILALC-ASTNAAFAPSVSQRTSVSVRESLDPTESMSEVEGAVKDAAPKVSDPFD  59
PtLHCX3      MKCIAAIALLA-T-TASAFNAF-----------------------GAAKKAA--PKKPVF  33
PtLHCX4      MKLFTIFLPLVLVGTAAGFAS------------------------GPFSK--KASPSPEV  34

CrLHCSR3     YTKNLPGVTAPFEGVFDPAGFLATASIKDVRRWRESEITHGRVAMLAALGFVVGEQLQDF  110
PtLHCX1      AKEDLVGAIPP-VGFFDPLGFADKADSPTLKRYREAELTHGRVAMLAVVGFLVGEAVEGS  90
PtLHCX2      SPRDLAGVVAP-TGFFDPAGFAARADAGTMKRYREAEVTHGRVGMMAVVGFLAGEAVEGS  118
PtLHCX3      SIETIPGALAP-VGIFDPLGFAAKADESTLKRYREAELTHGRVAMLATVGFLVGEAVEGS  92
PtLHCX4      SIESMPGIVAP-TGFFDPLRFAERAPSNTLKRYRECELTHGRVAMLATVGFLAGEAVQNT  93

CrLHCSR3     PLFFNWDGRVSGPAIYHFQQIGQGFWEPLLIAIGVAESYRVAVGWATPTGTGF---NSLK  167
PtLHCX1      S--FLFDASISGPAITHLSQVPAPFWVLLTIAIGASEQTRAVIGWVDPADAPVDKPGLLR  148
PtLHCX2      S--FLFDSQVSGPAITHLNQIPSIFWILLTVGIGASEVTRAQIGWVEPENVPPGKPGLLR  176
PtLHCX3      S--FLFDASIKGPAISHLAQVPTPFWVLLTIFIGAAEQTRAVIGWRDPSDVPFDKPGLLN  150
PtLHCX4      N--FLWNAQVSGPAITHIPQIPATFWVLLTLFIGVAELSRAQTAMVPPSDIPVGKAGRMR  151
             Region 1

CrLHCSR3     DDYEPGDLGFDPLGLKPTDPEELKVMQTKELNNGRLAMIAIAAFVAQELVEQTEIFEHLA  227
PtLHCX1      DDYVPGDLGFDPLGLKPSDPEELITLQTKELQNGRLAMLAAAGFMAQELVNGKGILENLQ  208
PtLHCX2      DDYVPGDIGFDPLGLKPSDAQALKSIQTKELQNGRLAMLAAAGCMAQELANGKGILENLG  236
PtLHCX3      EDYTPGDIGFDPLGLKPTDAEELRVLQTKELQNGRLAMLAAAGFMAQELVDGKGILEHLL  210
PtLHCX4      EDYNPGDIGFDPLNLMPESSEEFYRLQTKELQNGRLAMLGAAGFLAQEAVNGKGILENLF  211
                                                                Region 2

CrLHCSR3     LRFEKEAILELDDIERDLGLPVTPLPDNLKSL                    259
PtLHCX1      G-------------------------------                    209
PtLHCX2      L-------------------------------                    237
PtLHCX3      --------------------------------                    210
PtLHCX4      G-------------------------------                    212
```

B

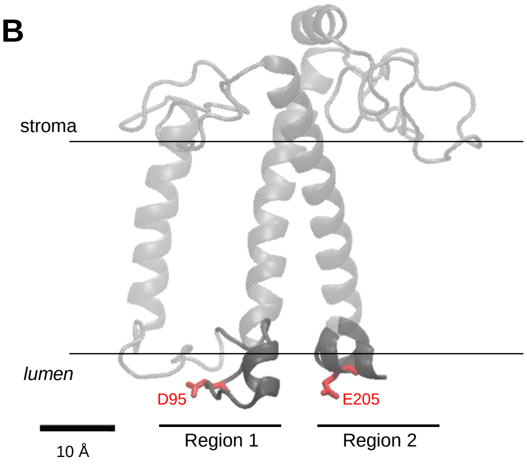

Fig. S2: Alignment of the LHCX proteins and three-dimensional model of the LHCX1. (A) Multiple sequence alignment of *Chlamydomonas reinhardtii* LHCSR3 and the four *Phaeodactylum tricornutum* LHCX proteins. Putative protonatable aminoacids in LHCX proteins conserved with respect to *C. reinhardtii* LHCSR3 (Ballottari et al. 2016) are indicated in red. (B) LHCX1 homology-based model from LHCII and CP29 crystallographic structures. Putative protonatable aminoacids conserved with respect to *C. reinhardtii* LHCSR3 are indicated in red (D95 and E205). Region 1 and 2 refers to part of LHCSR3 putatively exposed to thylakoydal lumen, where pH sensitive residues are located.

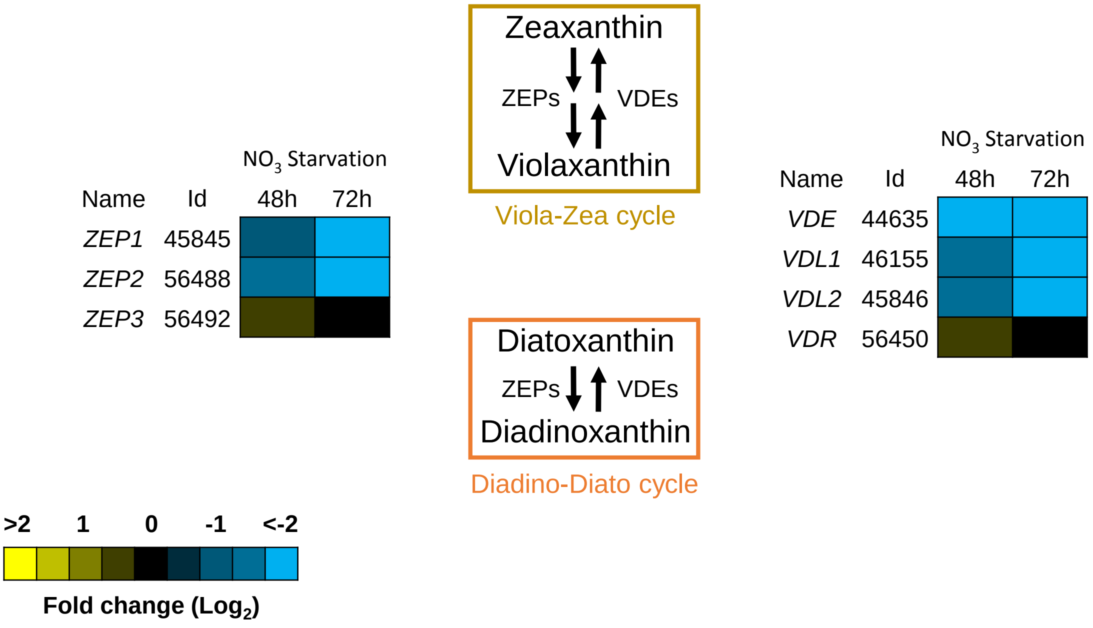

Fig. S3: Expression of *P. triconutum* xanthophyll cycles genes in nitrogen starvation. VDE (violaxanthin de-epoxidase), VDL1-2 (violaxanthin de-epoxidase like 1-2) and VDR (violaxanthin de-epoxidase related) are the enzymes putatively catalyzing the de-epoxidation reactions active in high light to form Zeaxanthin and Diatoxanthin from Violaxanthin and Diadinoxanthin, respectively. ZEP1, 2 and 3 (zeaxanthin epoxidase 1-2 and 3) enzymes putatively catalyse the epoxidation reaction, active in low light, to form Violaxanthin and Diadinoxanthin from Zeaxanthin and Diatoxanthin. Microarray data of cells after 48h and 72h of nitrogen starvation were taken from Alipanah *et al.*, 2015.

qPCR/PCR Oligos

Gene Id (Phatr2)	Oligo Name	Sequence (5' – 3')	Lenght
27278	Lhcx1Fw	CCTTGCTCTTATCGGCTCTG	20
	Lhcx1_Rv	ACGGTATCGCTTCAAAGTGG	20
56312	Lhcx2Fw	CAGCACTAATGCCGCTTTCG	20
	Lhcx2_Rv	CGTGAGTAACTTCCGCTTCC	20
44733	Lhcx3Fw	TCCCGTTGGTATCTTTGATCC	21
	Lhcx3_Rv	GAAGATCCTTCCACGGCTTC	20
38720	Lhcx4Fw	TCTTTGATCCACTCCGCTTC	20
	Lhcx4_Rv	GGCGTTCCATAGAAAGTTCG	20
10847	Rps F	CGAAGTCAACCAGGAAACCAA	21
	Rps R	GTGCAAGAGACCGGACATACC	21
34971	H4 Fw	AGGTCCTTCGCGACAATATC	20
	H4 Rv	ACGGAATCACGAATGACGTT	20

Cloning Oligos

Construct	Oligo Name	Sequence (5' – 3')	Lenght	Restriction Site
27278	L1OE_Fw	acgtGCGGCCGCATGAAGTTCGCTGCCACCATC	33	NotI
	L1OE_Rv	acgtGAATTCttaACCCTGAAGATTCTCAAGGA	33	EcoRI
56312	L2OE_Fw	acgtGCGGCCGCATGAAATTATCCTTGGCTATCC	34	NotI
	L2OE_Rv	acgtGAATTCttaGAGCCCAAGGTTTTCGAGGAT	34	EcoRI
44733	L3OE_Fw	acgtGCGGCCGCATGAAGTGCATCGCCGCTATC	33	NotI
	L3OE_Rv	acgtGAATTCttaGAGGAGGTGTTCCAAGATTCC	34	EcoRI
38720	L4OE_Fw	acgtGCGGCCGCATGAAATTGTTCACCATCTTC	33	NotI
	L4OE_Rv	acgtGAATTCttaGCCAAACAAATTCTCCAAAAT	34	EcoRI

Table S1: List of the oligonucleotides used in this work.
